# Tracking control of piezoelectric actuator using adaptive model

**DOI:** 10.1186/s40638-016-0039-x

**Published:** 2016-05-10

**Authors:** Tran Vu Minh, Nguyen Manh Linh, Xinkai Chen

**Affiliations:** School of Mechanical Engineering, Hanoi University of Science and Technology, Hanoi, Vietnam; Graduate School of Engineering and Science, Shibaura Institute of Technology, Saitama, 337-8570 Japan; Department of Electronic and Information Systems, Shibaura Institute of Technology, Saitama, 337-8570 Japan

**Keywords:** Piezoelectric actuator, Hysteresis, Adaptive model predictive control

## Abstract

Piezoelectric actuators (PEAs) have been widely used in micro- and nanopositioning applications due to their fine resolution, rapid responses, and large actuating forces. However, a major deficiency of PEAs is that their accuracy is seriously limited by hysteresis. This paper presents adaptive model predictive control technique for reducing hysteresis in PEAs based on autoregressive exogenous model. Experimental results show the effectiveness of the proposed method.

## Background

The use of piezoelectric actuator (PEA) has become very popular recently for a wide range of applications, including atomic force microscopes [1–3], adaptive optics [4], computer components [5], machine tools [6], aviation [7], internal combustion engines [8], micromanipulators [9] due to their subnanometer resolution, large actuating force, and rapid response. However, PEA exhibits hysteresis behavior in their response to an applied electrical energy. This leads to problems of inaccuracy, instability, and restricted system performance.

The control of PEA has been extensively studied recently. Ge and Jouaneh [10] discuss a comparison between a feedforward control, a regular PID control, and a PID feedback control with Preisach hysteresis. In this research, the nonlinear dynamics of piezoelectric actuator is first linearized and then reformulated the problem into a disturbance decoupling problem. In [11], an explicit inversion of Prandtl–Ishlinskii model is used to control a piezoelectric actuator. Webb et al. [12] proposed an adaptive hysteresis inverse cascade with the system, so that the system becomes a linear structure with uncertainties. Another adaptive control approach is fused with the Prandtl–Ishlinskii model without constructing a hysteresis inverse, since the inverse is usually difficult to be obtained [13]. In this concept, the implicit inversion of Prandtl–Ishlinskii model is developed and is associated with an adaptive control scheme. A new perfect inverse function of the hysteresis (which is described by Bouc–Wen model) is constructed and used to cancel the hysteresis effects in adaptive backstepping control design [14].

In this paper, the dynamics of the piezoelectric actuator is identified as a linear model with unknown parameters. These parameters will be updated online by using least square method. Then, a model predictive controller using estimated parameters is designed to achieve the desired control behavior. The experimental results show the effectiveness of the proposed method.

This paper is organized as follows. In “Modeling method” section, the adaptive model of PEA is given. In “Controlling method” section, the model predictive control design is presented. The experimental results are shown in “Result” section. “Discussion” section will conclude this paper.

## Modeling method

In this section, the dynamics of piezoelectric actuator can be identified as a linear model as follows

1 $$ m\textit{\"{y}}\left( t \right) + k\dot{y}\left( t \right) + cy\left( t \right) = u\left( t \right) $$

where *y*(*t*) denotes the position of piezoelectric actuator, *u*(*t*) is the force generated by PEA, *m* is the mass coefficient, *k* is the viscous friction coefficient of the PM, and *c* is the stiffness factor.

Now, express (1) as

2 $$ \frac{\text{d}}{{{\text{d}}t}}\left[ {\begin{array}{*{20}c} {y\left( t \right)} \\ {\dot{y}\left( t \right)} \\ \end{array} } \right] = \left[ {\begin{array}{*{20}c} 0\quad & 1 \\ { - \frac{c}{m}}\quad & { - \frac{k}{m}} \\ \end{array} } \right]\left[ {\begin{array}{*{20}c} {y\left( t \right)} \\ {\dot{y}\left( t \right)} \\ \end{array} } \right] + \left[ {\begin{array}{*{20}c} 0 \\ {\frac{1}{m}} \\ \end{array} } \right]u\left( t \right) . $$

Let T be the sampling period and suppose *y*(*t*) is constant during the sampling instant. By discretizing system (2), the input–output discrete time expression of system (1) can be given by

3 $$ y\left( k \right) = a\left( {q^{ - 1} } \right)y\left( {k - 1} \right) + b\left( {q^{ - 1} } \right)u\left( k \right) $$

where *q*^−1^ is the delay operator and *a*(*q*^−1^) and *b*(*q*^−1^) are polynomials defined by

4 $$ \begin{aligned} & a\left( {q^{ - 1} } \right) = - a_{1} - a_{2} q^{ - 1} \\ & b\left( {q^{ - 1} } \right) = b_{1} + b_{2} q^{ - 1} \\ \end{aligned} $$

The parameters *a*_1_, *a*_2_, *b*_1_, *b*_2_ are unknown.

Let *θ* be the vector of unknown system parameters

$$ \theta = \left[ {a_{1} ,\;a_{2} ,b_{1} ,\;b_{2} } \right]^{T} $$

Equation (2) can be written as

5 $$ y\left( k \right) = \phi^{T} (k - 1)\theta $$

where *φ*^*T*^(*k* − 1) = [*y*(*k* − 1), *y*(*k* − 2), *u*(*k* − 1), *u*(*k* − 2)].

Let $$ \hat{\theta }\left( k \right) = \left[ {\begin{array}{*{20}c} {\hat{\theta }_{1} \left( k \right)} & {\hat{\theta }_{2} \left( k \right)} & {\hat{\theta }_{3} \left( k \right)} & {\hat{\theta }_{4} \left( k \right)} \\ \end{array} } \right] $$ be the estimated of *θ*. Applying the least square method [15], the estimated parameters vector will be updated as follows

6 $$ \hat{\theta }\left( k \right) = \hat{\theta }\left( {k - 1} \right) + \frac{{P\left( {k - 1} \right)\phi \left( k \right)}}{{1 + \phi \left( k \right)^{T} P\left( {k - 1} \right)\phi \left( k \right)}}\left( {y\left( k \right) - \phi \left( {k - 1} \right)^{T} \hat{\theta }\left( {k - 1} \right)} \right) $$

7 $$ P\left( {k - 1} \right) = P\left( {k - 2} \right) - \frac{{P\left( {k - 2} \right)\phi \left( {k - 1} \right)\phi \left( {k - 1} \right)^{T} P\left( {k - 2} \right)}}{{1 + \phi \left( {k - 1} \right)^{T} P\left( {k - 2} \right)\phi \left( {k - 1} \right)}} $$

where *P*(*k*) is the covariance matrix with *P*(−1) is any positive define matrix *P*_0_. Usually, *P*_0_ is chosen as *P*_0_ = *λI*, where *λ* is a positive constant, *I* is the identity matrix.

## Controlling method

Using the estimated parameters, Eq. (3) can be rewritten as

8 $$ \begin{aligned} y\left( k \right) & = - \hat{\theta }_{1} (k - 1)y\left( {k - 1} \right) - \hat{\theta }_{2} (k - 1)y\left( {k - 1} \right) \\ & \quad + \hat{\theta }_{3} (k - 1)u\left( {k - 1} \right) + \hat{\theta }_{4} (k - 1)u\left( {k - 2} \right) \\ \end{aligned} $$

Defining *x*_1_(*k* + 1) = *x*_2_(*k*) = *y*(*k*), it gives

9 $$ \left\{ {\begin{array}{*{20}l} {x_{1} \left( {k + 1} \right) = x_{2} \left( k \right)} \hfill \\ {x_{2} \left( {k + 1} \right) = - \hat{\theta }_{1} (k)x_{2} \left( k \right) - \hat{\theta }_{2} (k)x_{1} \left( k \right) + \hat{\theta }_{3} (k)u\left( k \right) + \hat{\theta }_{4} (k)u\left( {k - 1} \right)} \hfill \\ \end{array} } \right. $$

Introducing new state variable *u*(*k*) = *u*(*k* − 1) + Δ*u*(*k*), Eq. (9) becomes

10 $$ \left[ {\begin{array}{*{20}c} {x_{1} \left( {k + 1} \right)} \\ {x_{2} \left( {k + 1} \right)} \\ {u\left( k \right)} \\ \end{array} } \right] = \left[ {\begin{array}{*{20}c} 0 \quad & 1\quad & 0\quad  \\ { - \hat{\theta }_{2} (k)}\quad & { - \hat{\theta }_{1} (k)}\quad & {\hat{\theta }_{3} (k) + \hat{\theta }_{4} (k)} \\ 0 \quad& 0 \quad& 1 \\ \end{array} } \right]\left[ {\begin{array}{*{20}c} {x_{1} \left( k \right)} \\ {x_{2} \left( k \right)} \\ {u\left( {k - 1} \right)} \\ \end{array} } \right] + \left[ {\begin{array}{*{20}c} 0 \\ {\hat{\theta }_{3} (k)} \\ 1 \\ \end{array} } \right]\Delta u\left( k \right) \; y\left( k \right) = \left[ {\begin{array}{*{20}c} 0\quad & 1\quad & 0 \\ \end{array} } \right]\left[ {\begin{array}{*{20}c} {x_{1} \left( k \right)} \\ {x_{2} \left( k \right)} \\ {u\left( {k - 1} \right)} \\ \end{array} } \right] $$

For simplicity, denote $$ M = \left[ {\begin{array}{*{20}c} 0\quad & \quad1 &\quad 0 \\ { - \hat{\theta }_{2} (k)} \quad& { - \hat{\theta }_{1} (k)}\quad & {\hat{\theta }_{3} (k) + \hat{\theta }_{4} (k)} \\ 0\quad & \quad0 &\quad 1 \\ \end{array} } \right] $$, $$ N = \left[ {\begin{array}{*{20}c} 0 \\ {\hat{\theta }_{3} (k)} \\ 1 \\ \end{array} } \right] $$ and $$ Q = \left[ {\begin{array}{*{20}c} 0 \quad& 1 \quad & 0 \\ \end{array} } \right] $$.

Introducing the cost function

11 $$ P = \sum\limits_{i = 1}^{{N_{p} }} {\omega \left( i \right)\left( {\hat{y}\left( {k + i|k} \right) - y_{d} \left( {k + i|k} \right)} \right)^{2} } + \sum\limits_{i = 1}^{{N_{p} }} {\rho \left( i \right)\left( {\Delta \hat{u}\left( {k + i|k} \right)} \right)^{2} } $$

where $\hat{y}\left( {k + i|k} \right)$ is the ith step predicted output from time *k*, *y*_*d*_(*k* + *i*|*k*) is the ith step reference signal from time k, $\Delta \hat{u}\left( {k + i|k} \right)$ is the difference between ith step predicted input from time *k* and control input at time k, *N*_*p*_ is the number of predicted steps, and *ω* and *ρ* are weighting coefficients.

In order to minimize the cost function (11), output predictions over the horizon must be computed. Predictive outputs can be obtained by using (10) recursively, resulting in:

12 $$ \hat{y}\left( {k + j} \right) = QM^{j} \hat{x}\left( k \right) + \sum\limits_{i = 0}^{j - 1} {QM^{j - i - 1} N \Delta u\left( {t + i} \right)} $$

Now, the predictions along the horizon are given by

13 $$ \hat{y}\left( k \right) = \left[ {\begin{array}{*{20}c} {\hat{y}\left( {k + 1|k} \right)} \\ {\hat{y}\left( {k + 2|k} \right)} \\ \vdots \\ {\hat{y}\left( {k + N_{p} |k} \right)} \\ \end{array} } \right] = \left[ {\begin{array}{*{20}c} {QM\hat{x}\left( k \right) + QN \Delta u\left( k \right)} \\ {QM^{2} \hat{x}\left( k \right) + \sum\limits_{i = 0}^{1} {QM^{1 - i} N \Delta u\left( {k + i} \right)} } \\ \vdots \\ {QM^{{N_{p} }} \hat{x}\left( k \right) + \sum\limits_{i = 0}^{{N_{p} - 1}} {QM^{{N_{p} - 1 - i}} N \Delta u\left( {k + i} \right)} } \\ \end{array} } \right] $$

For simplicity, define

14 $$ \hat{Y} = F\hat{x}\left( k \right) + H \Delta U $$

where $\hat{Y} = \left[ {\hat{y}\left( {k + 1|k} \right)\quad \hat{y}\left( {k + 2|k} \right) \ldots \hat{y}\left( {k + N_{p} |k} \right)} \right]^{T}$ is the predicted future output, $\Delta U = \left[ {\Delta u\left( k \right)\quad \Delta u\left( {k + 1} \right) \ldots \Delta u\left( {k + N_{p} - 1} \right)} \right]^{T}$ is the vector of future control increments, the matrix *H* defined as $$ H = \left[ {\begin{array}{*{20}c} {QN} & 0 & \cdots & \cdots & 0 \\ {QMN} & {QN} & \ddots & \ddots & \vdots \\ \vdots & \ddots & \ddots & \ddots & \vdots \\ {QM^{{N_{p} - 2}} N} & \ddots & \ddots & {QN} & 0 \\ {QM^{{N_{p} - 1}} N} & {QM^{{N_{p} - 2}} N} & \cdots & {QMN} & {QN} \\ \end{array} } \right] $$, and matrix *F* is defined as $$ F = \left[ {\begin{array}{*{20}c} {QM} & {QM^{2} } & \ldots & {QM^{{N_{p} }} } \\ \end{array} } \right]^{T} $$.

Consider the case where *ω*(*i*) = 1 and *ρ*(*i*) = *ρ*. The control sequence Δ*u* is calculated minimizing the cost function (10) that can be written as:

15 $$ P = \left( {H \Delta U + F\hat{x}\left( k \right) - Y_{d} } \right)^{T} \left( {H\Delta U + F\hat{x}\left( k \right) - Y_{d} } \right) + \rho \left( {\Delta U} \right)^{T} \left( {\Delta U} \right) $$

An analytical solution exists that can be calculated as follows

16 $$ \Delta U = \left( {H^{T} H + \rho I} \right)^{ - 1} H^{T} \left( {y_{d} - F\hat{x}\left( k \right)} \right) $$

It should be noted that only Δ*u*(*k*) is sent to the plant and all the computation is repeated at the next sampling time.

## Result

**Fig. 1:**
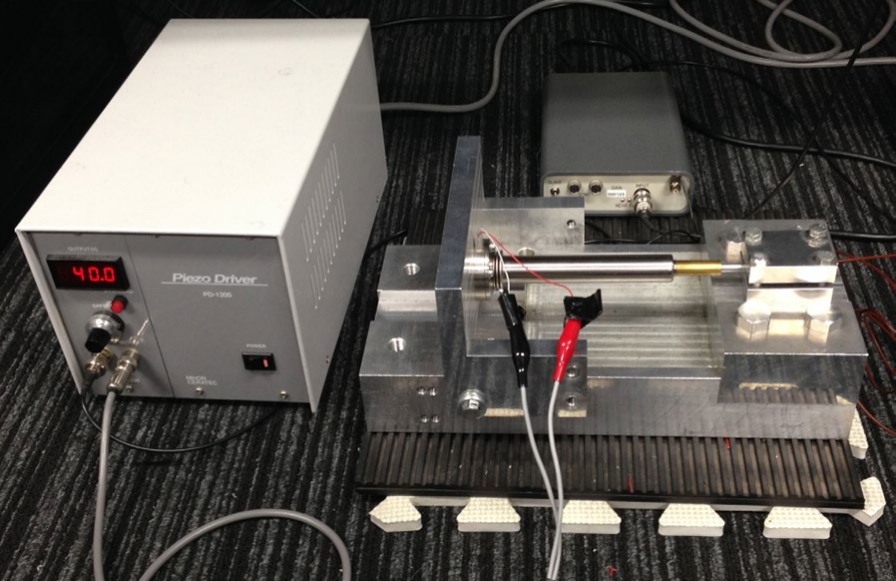
Experimental setup

**Fig. 2:**
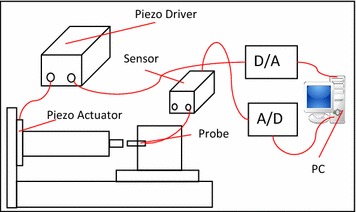
Experimental scheme

The experimental setup on piezoelectric actuator is shown in Fig. 1. Figure 2 shows the experimental scheme. The PEA is PFT-1110 (Nihon Ceratec Corporation). The specification of PFT 1110 is shown in. The displacement is measured by the noncontact capacitive displacement sensor (PS-1A Nanotex Corporation) which has 2-nm resolution. The experiments are conducted with 2 desired output s*y*_*d*1_(*k*) = 10 sin (2*π* × *k* × Δ*t*) μm and *y*_*d*2_(*k*) = 7 sin (2*π* × 5 × *k* × Δ*t*)+ 3 cos (2*π* × 0.5 × (1.5^−*k* × Δ*t*^) × *k* × Δ*t*) μm, where Δ*t* is sampling period and be chosen as 0.5 ms. The experiment results of proposed method are compared with those getting from PID controller.

Table 1 shows the experimental setting parameters.

**Table 1:** Experimental setting parameters

	*N*_*p*_	*ω*(*i*)	*ρ*(*i*)	*λ*	$\hat{\theta }\left( 0 \right)$	Offset (V)	Δ*t* (ms)
*y*_*d*1_(*k*)	3	1	0.1	0.1	0.2	30	0.5
*y*_*d*2_(*k*)	3	1	0.1	0.1	0.2	30	0.5

**Fig. 3:**
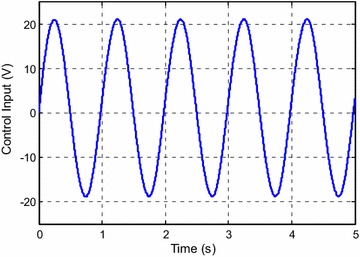
Control input for *y*_*d*1_(*k*)

**Fig. 4:**
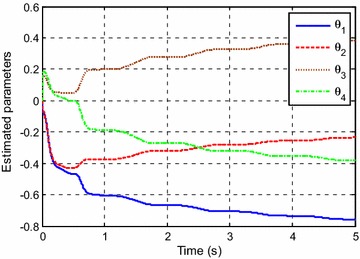
Estimated parameters for *y*_*d*1_(*k*)

Figure 3 shows the control input for the experiment with *y*_*d*1_(*k*). The estimated parameters are shown in Fig. 4.

**Fig. 5:**
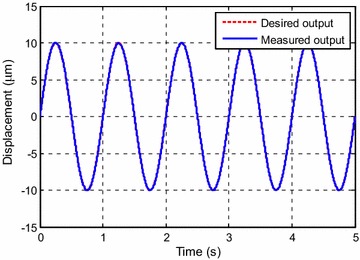
Tracking results for *y*_*d*1_(*k*)

**Fig. 6:**
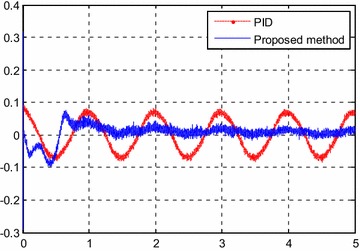
Tracking error for *y*_*d*1_(*k*)

Figure 5 shows the tracking result. The tracking error is shown in Fig. 6. It can be seen that the maximum error at steady state is about 0.4 %.

**Fig. 7:**
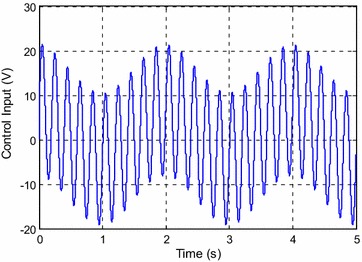
Control input for *y*_*d*2_(*k*)

**Fig. 8:**
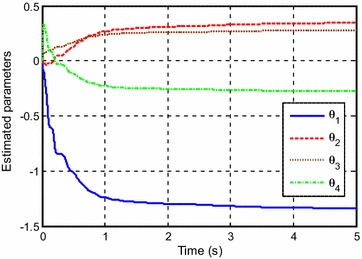
Estimated parameters for *y*_*d*2_(*k*)

Figure 7 shows the control input for the experiment with *y*_*d*2_(*k*). The estimated parameters are shown in Fig. 8.

**Fig. 9:**
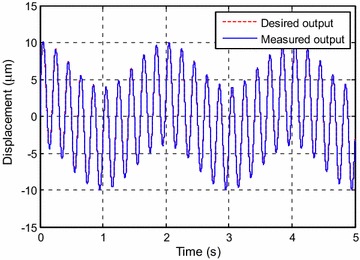
Tracking results for *y*_*d*2_(*k*)

**Fig. 10:**
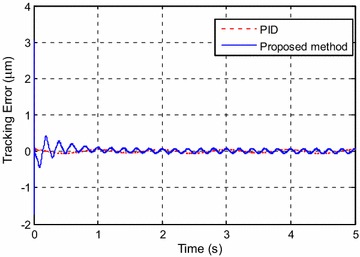
Tracking error for *y*_*d*2_(*k*)

Figure 9 shows the tracking result. The tracking error is shown in Fig. 10. It can be seen that the maximum error at steady state is about 1 %.

## Discussion

This paper has discussed the adaptive model predictive control for piezoelectric actuators, where the model of PEA is regarded as linear model. The unknown parameters in the model are estimated online. The proposed method shows its effectiveness in tracking performance. Moreover, it is simple and easy to be implemented. In the future, we will try to employ the proposed method to control piezo-actuated systems with load.
